# The Role of Lysosomes in a Broad Disease-Modifying Approach Evaluated across Transgenic Mouse Models of Alzheimer’s Disease and Parkinson’s Disease and Models of Mild Cognitive Impairment

**DOI:** 10.3390/ijms20184432

**Published:** 2019-09-09

**Authors:** Jeannie Hwang, Candice M. Estick, Uzoma S. Ikonne, David Butler, Morgan C. Pait, Lyndsie H. Elliott, Sarah Ruiz, Kaitlan Smith, Katherine M. Rentschler, Cary Mundell, Michael F. Almeida, Nicole Stumbling Bear, James P. Locklear, Yara Abumohsen, Cecily M. Ivey, Karen L.G. Farizatto, Ben A. Bahr

**Affiliations:** 1 William C. Friday Laboratory, Biotechnology Research and Training Center, University of North Carolina–Pembroke, Pembroke, NC 28372, USA; 2 Department of Pharmaceutical Sciences and the Neurosciences Program, University of Connecticut, Storrs, CT 06269, USA; 3 Department of Physiology and Neurobiology, University of Connecticut, Storrs, CT 06269, USA; 4 Center for Drug Discovery, Northeastern University, Boston, MA 02115, USA; 5 Department of Chemistry and Physics, University of North Carolina–Pembroke, Pembroke, NC 28372, USA; 6 Department of Biology, University of North Carolina–Pembroke, Pembroke, NC 28372, USA; 7 Molecular Biotechnology Program University of North Carolina–Pembroke, Pembroke, NC 28372, USA

**Keywords:** Aβ42, age, α-synuclein, cathepsin B, PADK, synaptic repair, ZFAD, Z-Phe-Ala-CHN_2_

## Abstract

Many neurodegenerative disorders have lysosomal impediments, and the list of proposed treatments targeting lysosomes is growing. We investigated the role of lysosomes in Alzheimer’s disease (AD) and other age-related disorders, as well as in a strategy to compensate for lysosomal disturbances. Comprehensive immunostaining was used to analyze brains from wild-type mice vs. amyloid precursor protein/presenilin-1 (APP/PS1) mice that express mutant proteins linked to familial AD. Also, lysosomal modulation was evaluated for inducing synaptic and behavioral improvements in transgenic models of AD and Parkinson’s disease, and in models of mild cognitive impairment (MCI). Amyloid plaques were surrounded by swollen organelles positive for the lysosome-associated membrane protein 1 (LAMP1) in the APP/PS1 cortex and hippocampus, regions with robust synaptic deterioration. Within neurons, lysosomes contain the amyloid β 42 (Aβ42) degradation product Aβ38, and this indicator of Aβ42 detoxification was augmented by Z-Phe-Ala-diazomethylketone (PADK; also known as ZFAD) as it enhanced the lysosomal hydrolase cathepsin B (CatB). PADK promoted Aβ42 colocalization with CatB in lysosomes that formed clusters in neurons, while reducing Aβ deposits as well. PADK also reduced amyloidogenic peptides and α-synuclein in correspondence with restored synaptic markers, and both synaptic and cognitive measures were improved in the APP/PS1 and MCI models. These findings indicate that lysosomal perturbation contributes to synaptic and cognitive decay, whereas safely enhancing protein clearance through modulated CatB ameliorates the compromised synapses and cognition, thus supporting early CatB upregulation as a disease-modifying therapy that may also slow the MCI to dementia continuum.

## 1. Introduction

Lysosomes, with their content of several classes of hydrolases, play a vital role in protein and glycoconjugate clearance for maintaining cellular homeostasis. Compromised clearing events by lysosomes are implicated in protein accumulation disorders including Alzheimer’s disease (AD), Parkinson’s disease, and Huntington’s disease, as well as in metabolic lysosomal storage disorders [1,2,3,4]. In addition, with regard to risk factors for the age-related neurodegenerative disorders, lysosomal instability is a feature of brain aging that influences the balance between protein synthesis and protein clearance [3,5,6,7], thus producing an impact on neuronal vulnerability in aged individuals.

Aging risk factors can lead to mild cognitive impairment (MCI) and different types of proteinopathies, including AD, which is the most common form of dementia, accounting for 60–80% of all cases of dementia. The protein accumulation stress contributing to AD pathogenesis is thought to stem from, at least in part, slower clearance rates for amyloid β (Aβ) peptides as compared to cognitively normal individuals [8]. Protein clearance mechanisms and related crosstalk vital for proteostasis were studied in different systems [9,10,11,12]. Most notably, cathepsin B (CatB), an Aβ42-degrading lysosomal protease [13,14,15], is particularly involved in compensatory events in response to multiple types of proteostatic stress. CatB upregulation events were found in response to the buildup of Aβ and related peptides [13,16], to huntingtin accumulation [17], and to the inhibition of protein clearing pathways [18,19]. Such response capability is likely involved in the increased number of lysosomes containing high levels of CatB in the at-risk neuronal populations of sporadic AD [20]. Interestingly, this potential compensatory response may explain the slow progression of AD that often occurs, as well as the AD proteinopathy that begins many years before cognitive symptoms become apparent [21]. CatB was in fact found to be increased in laser-captured cornu Ammonis 1 (CA1) neurons from Braak stage III AD brains [22], and its levels were also augmented in AD plasma samples [23,24]. Note that plasma CatB measures increased with exercise in humans and monkeys [25], and the resulting CatB levels correlated with improved memory. While previous studies suggested some neuroprotective agents acted by inhibiting CatB, evidence points to such protectants as acting through a non-CatB pathway [26,27], in contrast to CatB-enhancing properties found linked to synaptic and neuronal repair. The CatB proteolytic responses support early and prolonged signaling events that work to maintain proteostasis and reduce pathogenic protein deposition and, when effective, they perhaps explain the extended nature of AD in many individuals.

In this study, lysosomes were examined for their role in protein accumulation pathology found in amyloid precursor protein/presenilin-1 (APP/PS1) double transgenic mice, an AD mouse model with a link to lysosomal dysfunction and that exhibits Aβ deposition in correspondence with synaptic and cognitive declines [14,28,29]. A strategy to offset lysosomal perturbation through CatB modulation was also tested for promoting recovery of synapse integrity and cognitive functions. Since recent warnings indicate that overexpression of human APP produces phenotypes unrelated to AD [30], the current study also examined the CatB modulatory approach in two rodent models of age-related cognitive impairment.

## 2. Results

### 2.1. Assessment of Protein Accumulation Pathology in the APP/PS1 Transgenic Mouse Model of AD

#### 2.1.1. Immunostaining Aβ Species, Synaptic Markers, and Lysosomal-Associated Membrane Protein 1 (LAMP1)

**Figure 1:**
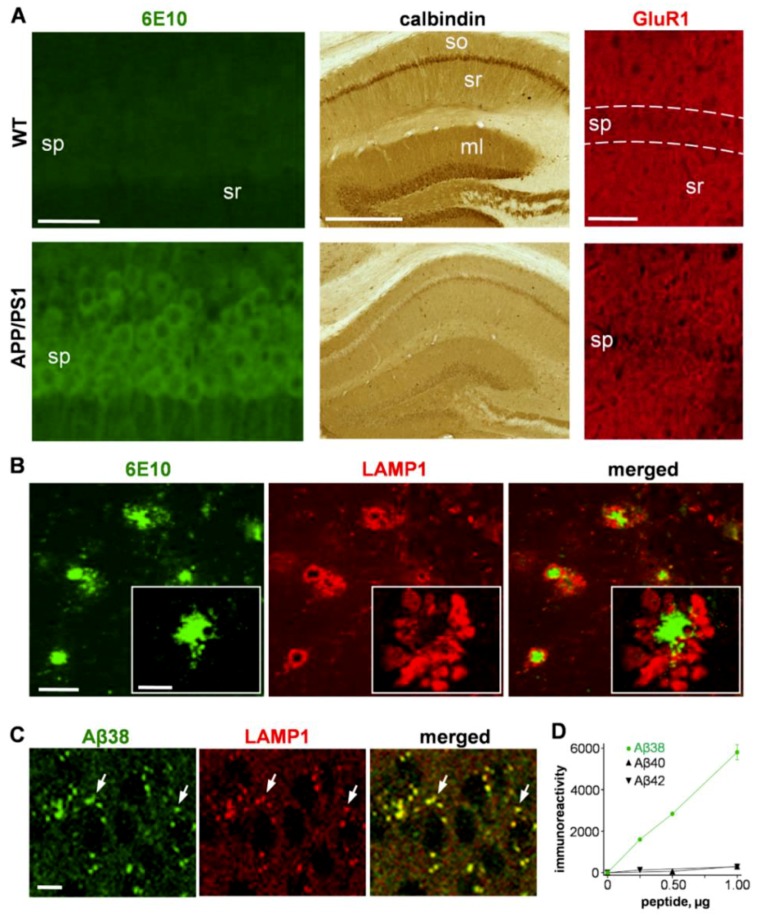
Proteinopathy in amyloid precursor protein/presenilin-1 (APP/PS1) transgenic mice is linked to alterations in neuronal lysosomes**.** Fixed tissue sections from 22–23 month-old APP/PS1 mice and age-matched wild-type littermates (WT) were immunostained with the 6E10 antibody to amyloid β 1–16 (Aβ1–16; green; size bar: 30 µm), anti-calbindin (middle; size bar: 500 µm), and anti-glutamate receptor 1 (GluR1; red; size bar: 50 µm) to monitor accumulation of potentially amyloidogenic material and synaptic pathology (**A**). ml, molecular layer; so, stratum oriens; sp, stratum pyramidale; sr, stratum radiatum. APP/PS1 hippocampal tissue was also double-labeled and images were captured of 6E10-positive deposits (green) surrounded by lysosome-associated membrane protein 1 (LAMP1) staining (red), indicating severely swollen lysosomes accumulated at amyloid plaques (**B**, size bar: 50 µm; insert size bar: 10 μm). In immunostained transgenic tissue, Aβ38 (green) and LAMP1 (red) were colocalized in lysosomes (**C**; size bar: 10 µm) found within hippocampal neurons (see arrows). To confirm the specificity of the anti-Aβ38 antibody, increasing amounts of three Aβ peptides were assessed by an ELISA protocol using anti-Aβ38 for detection (**D**).

To assess protein accumulation pathology related to AD, immunostaining procedures were applied to brain tissue from APP/PS1 mice that express mutant forms of APP and presenilin-1 and exhibit progressive amyloid deposition and neuritic abnormalities [31]. Hippocampal tissue stained with the 6E10 antibody to human Aβ exhibited neuronal labeling in APP/PS1 sections but not in littermate controls, and the staining of synaptic markers calbindin and AMPA-type glutamate receptor subunit 1 (GluR1) was reduced in the transgenic mice (Figure 1A). The Aβ pathology in the APP/PS1 hippocampus and cortex included deposits of 40–90 µm in approximate diameter, and, as previously described [32,33], the 6E10-positive plaques (green) were surrounded by varying levels of large, organelle-type structures stained for the lysosome-associated membrane protein 1 (LAMP1; red), a specific marker that shuttles between lysosomes, late endosomes, and the plasma membrane (see Figure 1B). Note that the larger plaques were clearly associated with more of the swollen LAMP1-positive structures than smaller plaques.

#### 2.1.2. Colocalization of LAMP1 and the Aβ42 Degradation Product Aβ38 in Neurons

Next, we used a double immunofluorescence procedure to help identify the location of Aβ42 detoxification events in confocal images. In contrast to the abnormal LAMP1-containing structures that accumulate around amyloid plaques in the neuropil, much smaller LAMP1-positive organelles were found within pyramidal neurons, corresponding to individual lysosomes that contain the Aβ42 degradation product Aβ38 (Figure 1C). The colocalized immunofluorescence staining was determined with anti-LAMP1 along with an antibody that selectively recognizes the less pathogenic Aβ38 peptide (Figure 1D) as compared to no detection of Aβ40 and Aβ42 peptides in tests using Aβ38 sandwich ELISA.

### 2.2. Positive CatB Modulation as a Compensatory Strategy in APP/PS1 Transgenic Mice

#### 2.2.1. CatB Enhancement by Z-Phe-Ala-Diazomethylketone (PADK) is Associated with Improved Aβ42 Detoxification in Lysosomes

**Figure 2:**
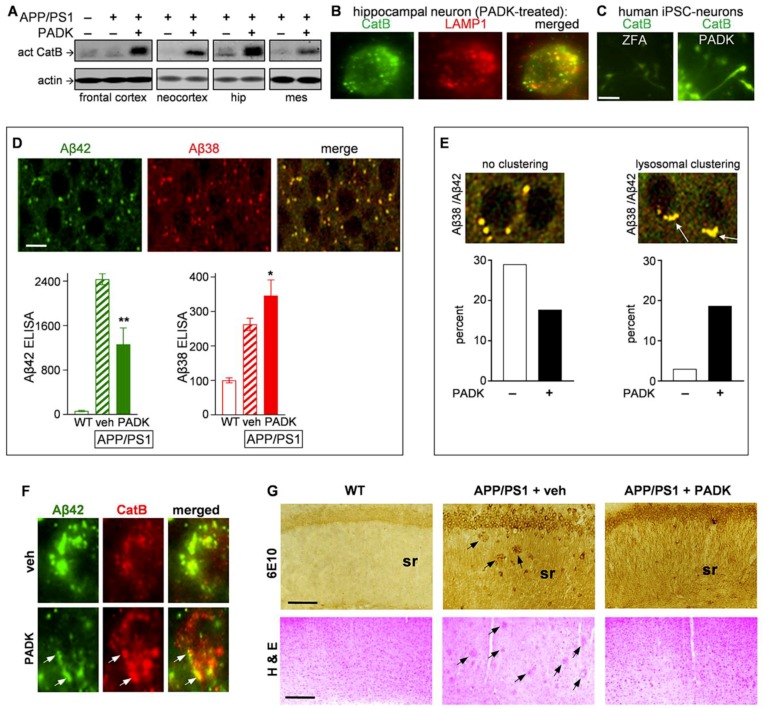
Enhancement of cathepsin B (CatB) promotes a lysosomal pathway for Aβ42 detoxification and clearance. Equal protein aliquots of homogenates from vehicle (−) and Z-Phe-Ala-diazomethylketone (PADK)-treated APP/PS1 mice (+) in cropped immunoblots were assessed for the 30-kDa active CatB isoform alongside wild-type controls (**A**) (also see immunoblots in Supplementary Materials). hip, hippocampus; mes, mesencephalon. Neurons in PADK-treated mice were double-labeled for CatB (green) and LAMP1 (red); an example is shown with lysosomal localization of the modulated CatB (**B**; view-field width: 15 µm). Cultures of human induced pluripotent stem cell (iPSC)-derived neurons were treated for three days with 5 μM inactive Z-Phe-Ala-OH (ZFA) compound or with PADK, followed by fixation and CatB staining (**C**; size bar: 50 µm). APP/PS1 hippocampal tissue was double-labeled with anti-Aβ42 (green) and anti-Aβ38 antibodies (red), showing striking correspondence in the merged image (**D**; size bar: 10 µm). The two antibodies were also used to quantify the distinct peptides (fmol/mg protein) in samples from the different animal groups with selective sandwich ELISA protocols. Nonparametric Mann–Whitney tests compared to vehicle-treated APP/PS1 data: * *p* < 0.05, ** *p* = 0.01. Merged confocal images of anti-Aβ42 and anti-Aβ38 immunostaining (**E**) show representative neurons with no apparent lysosomal clustering (left) vs. neurons with polar accumulation of clustered lysosomes (right). Individual pyramidal neurons from vehicle- (*n* = 100) and PADK-treated tissue (*n* = 150) were categorized to determine the percentage of neurons with no clustering (left graph) vs. the percentage with clustering of multiple lysosomes (right graph). χ^2^ analysis of categorical distributions: χ^2^ = 27.1, *p* < 0.0001. The vehicle- and PADK-treated tissue was also double-labeled to assess the increase in organellar CatB (red) and associated decrease in anti-Aβ42 staining (green) within pyramidal neurons (**F**), with arrows denoting colocalization found in clustered organelles exhibiting polar distribution (view-field: 15 µm). Hippocampal sections from wild-type mice and vehicle- vs. PADK-treated transgenics were assessed for 6E10 labeling (**G**, upper panels) to assess the level of amyloid plaques (see arrows; size bar: 100 µm). Cortical sections from the three animal groups were subjected to hematoxylin–eosin staining (G, lower panels; size bar: 150 µm). sr, stratum radiatum; veh, vehicle.

To target the role of lysosomes in Aβ42 detoxification as a putative therapeutic approach, we used Z-Phe-Ala-diazomethylketone (PADK; 18 mg/kg/day, 11 days; intraperitoneally (i.p.)) to increase CatB, a cathepsin family member that proteolytically cleaves Aβ42 into less amyloidogenic peptides [13,34] and produces the shorter Aβ38 shown to correspond with Aβ42 clearance in AD transgenic mice [14]. After 11 daily injections into 22–23-month-old APP/PS1 mice, PADK produced marked increases in the active form of CatB across regional immunoblot samples, as compared to vehicle-injected mice (Figure 2A). Densitometric quantification of the CatB immunoblots, using only those samples that fell within the linear range of detectable measures, found that active CatB was increased 6–9-fold among the cortical samples, 5–8-fold in hippocampal samples, and 5–6-fold in the mesencephalic samples. The modulated CatB was confirmed as being localized to lysosomes, in which pyramidal neurons from the brains of PADK-treated mice exhibited intense CatB immunostaining that co-localized with LAMP1-positive organelles (Figure 2B). The number of LAMP1-positive lysosomes in neurons was unchanged by PADK as previously found [14], and the extent of enhancement of the CatB isoform correlated with increased levels of CatB enzymatic activity across a group of samples (*R* = 0.894; *p* < 0.001). The CatB immunoreactivity in human induced pluripotent stem cell (iPSC)-derived neurons was also increased by PADK (Figure 2C) as compared to those treated with the inactive control compound Z-Phe-Ala-OH (ZFA). Both the human neurons and rat cultures exhibited EC_50_ values for PADK in the 3–5 μM range for the positive CatB modulation.

The lysosomal modulation by PADK also decreased Aβ42 levels (see green bar graph in Figure 2D; Kruskal–Wallis nonparametric test: *p* = 0.010) while levels of the Aβ38 peptide were increased (red bar graph; Kruskal–Wallis test: *p* = 0.011), as determined by selective sandwich ELISA protocols. Both peptides were increased in the APP/PS1 brain, and their opposing PADK effects indicate that Aβ42 detoxification is mediated by lysosomes, as suggested previously [13,14] and further supported by the colocalization of Aβ42 and Aβ38 in small organelles in neurons (see immunohistochemistry images in Figure 2D). Perhaps related to the lysosomal truncation of Aβ42, PADK’s influence on lysosomes includes the induction of their polar clustering within neurons (Figure 2E). This deduction resulted from assessing individual hippocampal neurons from the vehicle- and PADK-treated brains (*n* = 100 and 150 neurons, respectively), with PADK reducing the relative number of neurons lacking clustered lysosomes positive for both Aβ42 and Aβ38 (left graph), while increasing the percentage of those neurons exhibiting lysosomal clustering (right graph; χ^2^ analysis: *p* < 0.0001). Related to the clustering effect, as well as to Aβ42 detoxification in lysosomes, PADK was found to also elicit polar organellar clustering in pyramidal neurons, with the lysosomes exhibiting colocalized staining of CatB and Aβ42 (see arrows in Figure 2F). Note the evident reduction of intracellular Aβ42 accumulation by PADK in connection with increased CatB staining, with several CatB-positive lysosomes involved in proteolytic detoxification.

The findings indicating enhancement of Aβ42 truncation and, thus, detoxification by PADK were further substantiated by PADK’s ability to promote clearance of Aβ deposition in APP/PS1 brain sections (Figure 2G, upper panels). The area of 6E10 deposit staining in the CA1 dendritic field, averaged across three adjacent 200 × 200 μm regions of interest per APP/PS1 mouse (4490 ± 1109 μm^2^, mean ± SEM, *n* = 10 vehicle-treated mice), was reduced by 80% in the PADK-treated mice (755 ± 130 μm^2^, *n =* 10; nonparametric Mann–Whitney test: *p* = 0.0052). The latter measures used tissue sections with neuronal densities and stratum pyramidale areas that closely compared to those in the vehicle-treated group (98.8 ± 6.7%). PADK also caused a reduction in the degree of plaque staining in the frontal cortex (not shown). In addition to the clearance of 6E10 immunoreactive material, extracellular deposits detected in cortical layers 2–5 with hematoxylin–eosin staining were also reduced by PADK (lower panels of Figure 2G).

#### 2.2.2. Positive CatB Modulator Reduces Protein Accumulation Events and Improves Synaptic and Cognitive Measures

**Figure 3:**
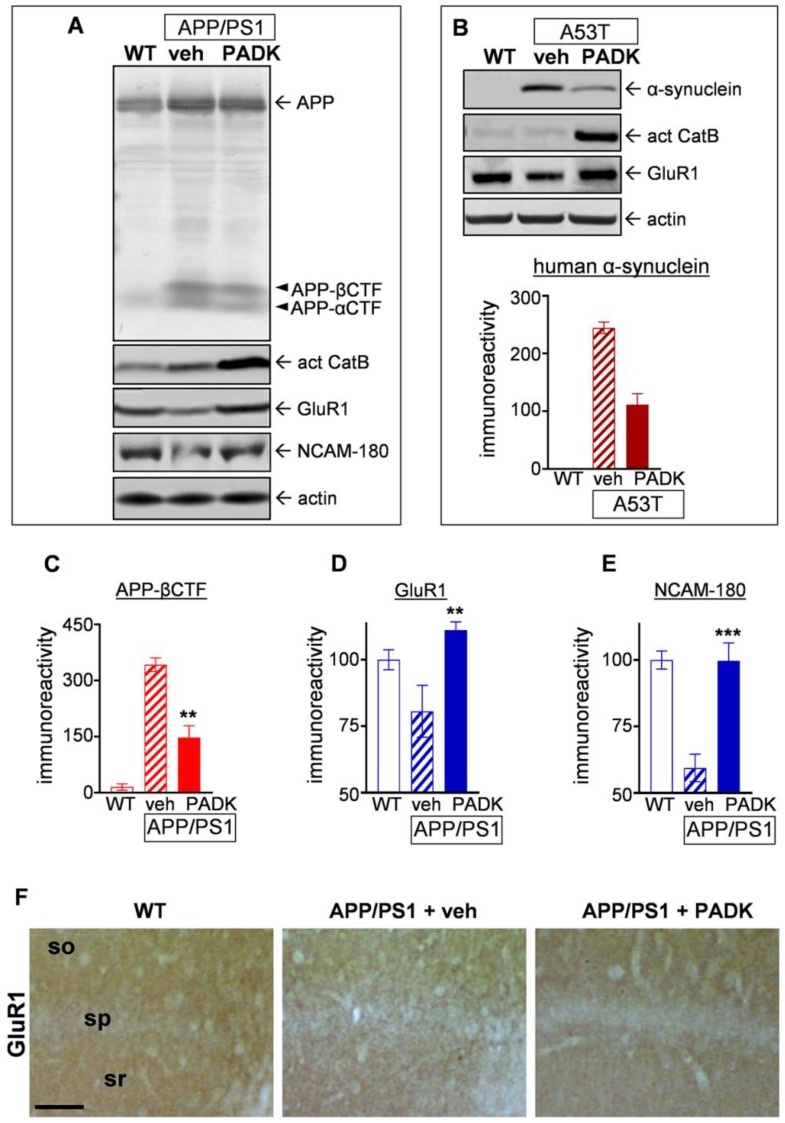
Positive CatB modulator PADK reduces distinct protein accumulation events in association with the recovery of synaptic markers in hippocampus. Equal protein aliquots of brain samples from wild-type mice (WT) and from APP/PS1 mice treated with either vehicle or PADK (18 mg/kg daily for 11 days; intraperitoneally (i.p.)) are shown in cropped immunoblots that were assessed for the α-cleaved and β-secretase-derived carboxyterminal APP fragments (APP-αCTF and APP-βCTF), active CatB isoform (act CatB), synaptic markers GluR1, neural cell adhesion molecule (NCAM)-180, and actin for a load control (**A**) (see immunoblots in Supplementary Materials). Brain homogenate samples from vehicle- and PADK-treated A53T Parkinson’s disease transgenic mice (18 mg/kg, 11 days; i.p.), in cropped immunoblots, were similarly assessed alongside control samples, to label human α-synuclein, active CatB, GluR1, and actin (**B**) (the immunoblots are also presented in Supplementary Materials), and integrated optical density measures for the labeled α-synuclein band are shown (Kruskal–Wallis test: *p* < 0.01). To evaluate vehicle- vs. PADK-treated APP/PS1 samples, the immunoreactivity measures for APP-βCTF were plotted (**C**), along with the GluR1 (**D**) and NCAM-180 (**E**) measures normalized to their respective levels in wild-type animals. Unpaired, nonparametric Mann–Whitney tests compared to the vehicle-treated transgenic group: ** *p* <0.01, *** *p* < 0.001. The AMPA receptor subunit GluR1 immunostaining was assessed in field CA1 (**F**, bar size: 100 µm). so, stratum oriens; sp, stratum pyramidale; sr, stratum radiatum.

In addition to reducing Aβ42 and plaque levels in APP/PS1 mice, Figure 3A shows that PADK’s induction of active CatB is also linked to increased clearance of the β-secretase-derived carboxyterminal fragment of APP (APP-βCTF), an amyloidogenic species shown to accumulate in CatB- and LAMP1-positive organelles in the triple-transgenic (3×Tg) LaFerla mouse model of AD [35]. This enhanced clearance was associated with improved levels of synaptic markers (Figure 3A), as also found associated with reduced levels of α-synuclein in the A53T transgenic mouse model of Parkinson’s disease (Figure 3B). The α-synuclein results were significant as per the Kruskal–Wallis test (*p* < 0.01), and, while the post hoc test only approached significance, the *t*-test resulted in *p* = 0.0286 when comparing the PADK group to the vehicle-treated transgenic mice. In addition, note that the within-animal analysis found a positive relationship between percent reduction in α-synuclein and the recovery level of α-amino-3-hydroxy-5-methyl-4-isoxazolepropionate (AMPA) receptor subunit GluR1 as determined by linear regression (*p* = 0.039). Due to the evident clearance of α-synuclein and APP-βCTF mediated by lysosomal modulation, it was not unexpected for the α-cleaved carboxyterminal APP fragment (APP-αCTF) to also exhibit improved clearance in PADK-treated mice (Figure 3A).

**Figure 4:**
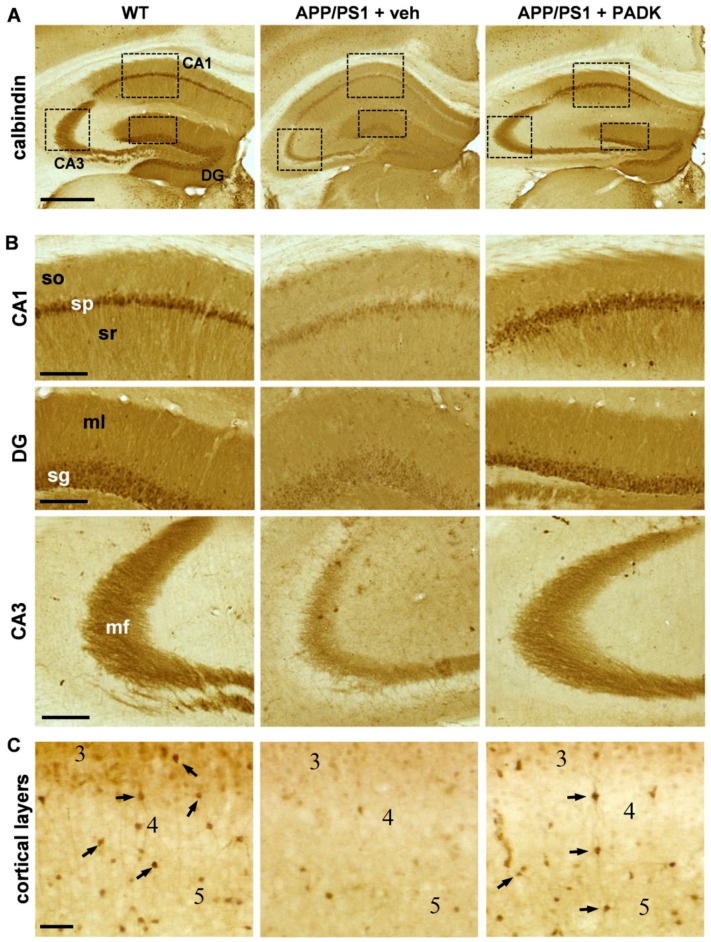
APP/PS1 transgenic mice treated with PADK exhibit restored calbindin immunostaining. Wild-type mice (WT) alongside vehicle- and PADK-treated APP/PS1 mice were assessed by immunohistochemistry in order to visualize calbindin D28k distribution in the hippocampus (**A**; size bar: 500 µm). The boxed areas of the different subfields in A are shown at higher magnification (**B**; size bar: 100 µm), as are interneurons (arrows) immunolabeled in cortical layers 3–5 (**C**; bar size: 50 µm). DG, dentate gyrus; mf, mossy fibers; ml, molecular layer; sg, stratum granulosum; so, stratum oriens; sp, stratum pyramidale; sr, stratum radiatum.

The striking APP-βCTF levels generated in APP/PS1 brains corresponded with decreases in GluR1 (Mann–Whitney test: *p* = 0.013) and the 180-kDa neural cell adhesion molecule (NCAM-180; Mann–Whitney test: *p* < 0.0001), and PADK treatment reduced APP-βCTF measures by 59% (Figure 3C; Mann–Whitney test: *p* = 0.0079) while also eliciting recovery of the synaptic markers. In fact, PADK’s modulatory effect completely restored GluR1 (Figure 3D; Mann–Whitney test: *p* = 0.0053) and NCAM-180 levels (Figure 3E; Mann–Whitney test: *p* = 0.0002) to those found in wild-type mice. It is noteworthy that the reduced dendritic labeling of GluR1 in the APP/PS1 hippocampus also exhibited restoration by PADK (Figure 3F). Further indications of PADK restoring neuropil integrity were found with immunohistochemical staining of calbindin-D28k across the hippocampal subfields (Figure 4A), and this is of particular interest since calbindin depletion is a strong correlate of cognitive decline [13,36,37,38]. Renewal of calbindin, a calcium-binding protein important for neuronal survival, was clearly evident in dendritic fields and mossy fibers (Figure 4B), as well as among cortical interneurons (see arrows in Figure 4C).

**Figure 5:**
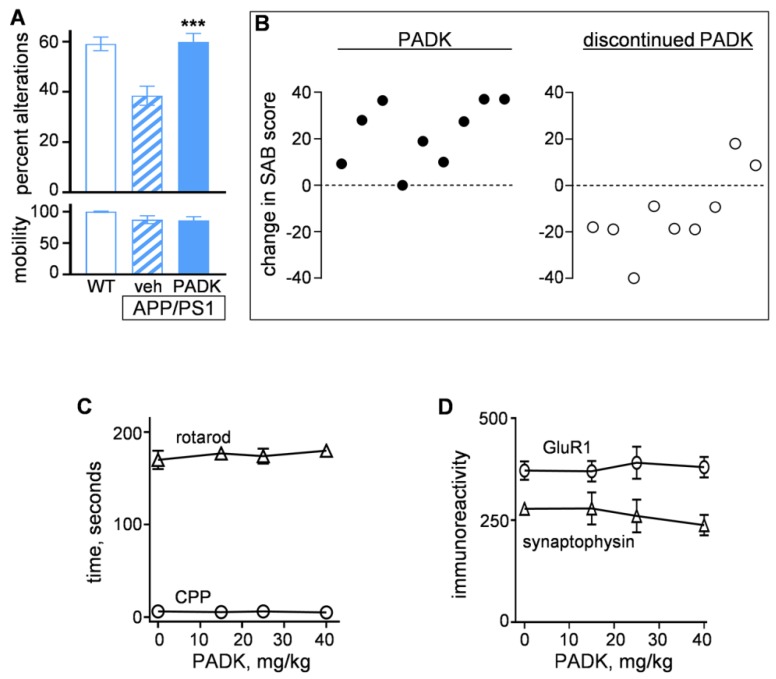
CatB enhancer elicits functional recovery in APP/PS1 mice. Age-matched, wild-type mice (WT; *n* = 29) and APP/PS1 mice treated with vehicle (*n* = 19) vs. PADK (18 mg/kg, 11 days) (*n* = 19) were tested for spontaneous alternation behavior (SAB) in a T-maze (**A**). Mann–Whitney test compared to vehicle-treated transgenics: *** *p* < 0.001. Mobility during maze exploration is shown in the lower graph. Percent changes in SAB are shown for a subset of the PADK-treated APP/PS1 mice compared to the mean SAB of vehicle-treated APP/PS1 mice (B, left graph). The same subset of PADK-treated mice was assessed for SAB three weeks after PADK treatment was discontinued, and the percent change was plotted as compared to the respective scores determined with PADK treatment (**B**, right graph) (Mann–Whitney test compared to data in left graph: *p* < 0.001). As part of central nervous system (CNS) safety evaluation, 9–10-month-old mice received 0–40 mg/kg PADK (i.p.) daily for nine days, followed by the rotarod test and a conditioned place preference (CPP) measure for entering previously trained compartment of reward placement (**C**). Hippocampal tissue from the mice was assessed by immunoblot for synaptic markers (**D**).

The protection of synapses by positive CatB modulation is of principal importance in light of synaptic decline being one of the best correlates of cognitive dysfunction in dementias. Indeed, the different CatB-enhancing compounds identified exhibit a consistent characteristic of inducing recovery of the synaptic marker GluR1 (Table 1). To determine if the improved protein clearance and synaptic recovery found in PADK-treated APP/PS1 mice translate to functional rescue, we evaluated the behavioral performance measures from those animals. The vehicle-treated APP/PS1 mice exhibited 35% reduction in spontaneous alternation behavior (Mann–Whitney nonparametric test: *p* < 0.0001), consistent with episodic memory deficits reported for this transgenic AD model [14,28]. As predicted, due to PADK-mediated restoration of synaptic markers in neuronal networks important for learning and memory, the CatB modulator led to complete recovery of T-maze alternation behavior (Figure 5A; Mann–Whitney test: *p* = 0.0002). No difference in exploratory mobility in the maze was evident among the groups of mice (lower graph in Figure 5A). In addition to PADK being linked to cognitive repair, SD1002 from Table 1 also exhibited 83% functional recovery in the AD mouse model (Mann–Whitney test: *p* = 0.033), and E64d is a compound shown by several studies to improve cognition [27]. From the PADK results, it is noteworthy that a within-animal analysis between percentage increase in hippocampal CatB and the extent of functional recovery found a positive relationship as determined by linear regression (*R* = 0.860; *p* < 0.001).

To properly address whether CatB-enhancing molecules represent a disease-modifying approach to treat CNS disorders, it is important to test if such a compound exhibits the essential druglike property of having reversible functional effects upon discontinuance of its administration. In Figure 5B, the majority of SAB scores from a group of PADK-treated APP/PS1 mice exhibited improvements in alternation behavior (left graph), whereas the majority of the same mice subsequently declined in the SAB performance three weeks after PADK treatment was ceased (right graph; *p* = 0.0005 compared to left graph). Such reversal corresponded with the 3.5–4.5-fold cathepsin modulation returning to 100–114% of control levels. In addition to reversibility, other druglike properties were evaluated (Table 2), with PADK-treated APP/PS1 mice demonstrating selectivity for modifying CatB activity as there were no indications of altered lysosomal biogenesis or gene expression. No change in the proteasome system was evident, and levels of the CatB-interacting protein, sushi repeat-containing protein X-linked 2 (SRPX2), were also unchanged. However there was a trend for SRPX2 being reduced in concentration as CatB was upregulated in PADK-treated mice.

From initial tests for safety, no weight changes were found in the different groups of aged mice in the study (Table 2), including among female mice that exhibited the same 3–4-fold increase in CatB as male littermates. Central nervous system (CNS) safety was further evaluated in middle-aged mice treated with 0–40 mg/kg PADK for nine days, with the manifold lysosomal modulation plateauing at near 25 mg/kg, far below the 250–500 mg/kg range for other indications. No changes in mobility, balance and coordination, or memory performance were found across the dosages (Figure 5C), and pre- and postsynaptic markers were also unchanged (Figure 5D).

### 2.3. Assessment of Positive CatB Modulation as a Potential Preventive Strategy in Models of Age-Related Cognitive Impairment

#### 2.3.1. CatB Enhancement by PADK for Synaptic and Functional Improvements in Aged Female Fischer Rats

**Figure 6:**
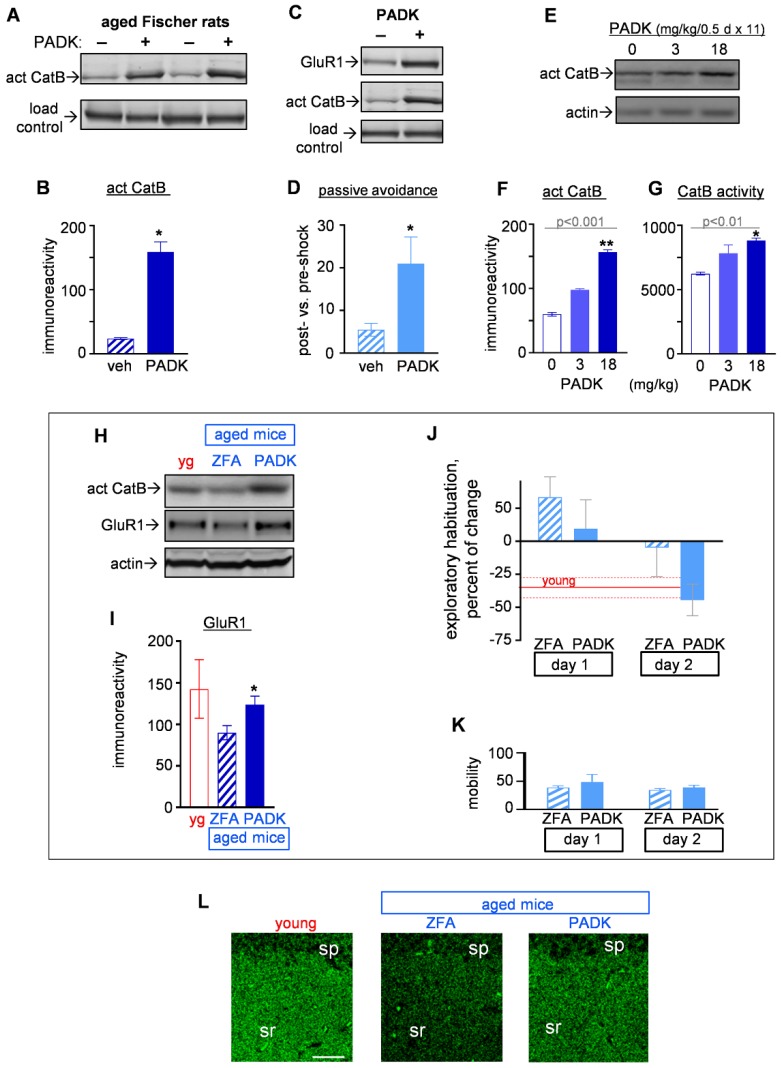
Synaptic and cognitive effects produced by the positive CatB modulator PADK in models of mild cognitive impairment (MCI). Equal protein aliquots of hippocampal homogenates from vehicle- and PADK-treated female Fischer rats are shown in cropped immunoblots that were assessed for active CatB (**A**; see Supplementary Materials) which was quantified by integrated optical density measures (**B**; Mann–Whitney test: * *p* < 0.05). Fischer rat samples in cropped immunoblots were also stained for GluR1 in parallel with CatB (**C**; see Supplementary Materials). The groups of Fischer rats were tested for passive avoidance, and those treated with PADK exhibited a more pronounced level of learning indicated from the ratio of post-shock/pre-shock latency times (**D**; Mann–Whitney test: * *p* < 0.05). For oral dosing, Sprague-Dawley rats received 20 mg/kg inactive ZFA (designated as 0 PADK) or 3–18 mg/kg PADK twice daily for 11 days, and brain samples were subsequently assessed as shown in cropped immunoblots for active CatB (**E**; also see Supplementary Materials). Integrated optical densities of the active CatB isoform exhibit a dose-dependent increase (**F**) (Kruskal–Wallis test: *p* < 0.001; post hoc test compared to the ZFA control group: ** *p* = 0.01), as do the measures of CatB activity (**G**) (Kruskal–Wallis test: *p* < 0.01; post hoc test compared to the ZFA group: * *p* < 0.05). Sample aliquots from orally dosed young (yg) and vehicle- vs. PADK-treated 22-month mice are shown in cropped immunoblots stained for active CatB, GluR1, and actin (**H**; see Supplementary Materials), and GluR1 levels were significantly increased by PADK (**I**; Mann–Whitney test: * *p* < 0.05). Prior measures of exploratory habituation by the mice (**J**) show the young group with reduced exploration on day two (mean ± dotted standard error of the mean (SEM) in red; *p* = 0.0156) and PADK treatment leading to similar habituation behavior in the aged mice (Kruskal–Wallis test: *p* = 0.0115). Mobility units from initial minutes of each exploring period are shown (**K**). The mouse brains were immunostained for synaptophysin in hippocampal CA1 (**L**). Size bar: 50 µm. sp, stratum pyramidale; sr, stratum radiatum.

It was important to include animal models without overexpression of human APP to assess lysosomal enhancement as a compensatory strategy against synaptic and behavioral deficits. Since middle-aged Fischer rats were listed as a model of mild cognitive impairment (MCI) with age-related declines in cognition and synaptic markers [39,40,41], we added them to the testing of PADK-mediated lysosomal enhancement. PADK treatment via i.p. injections led to increased levels of active CatB in the brains of the 11-month-old rats (Figure 6A), and hippocampal samples were found to exhibit a seven-fold increase in the CatB isoform (Figure 6B). Evidence of enhanced GluR1 levels in the PADK-treated Fischer rats corresponded with both the CatB upregulation (Figure 6C) and improved performance on the passive avoidance task (Figure 6D). No difference in overall mobility was evident between vehicle and PADK groups (*p* = 0.83). Note that the 11-month female Fischer rats treated with vehicle exhibited a deficit in the shock-induced passive avoidance learning as compared to young three-month-old rats (Wilcoxon test: *p* = 0.0042), whereas PADK-treated aged rats did not (*p* = 0.187) due to their significantly increased post-shock latency time for entering the avoidance chamber.

#### 2.3.2. CatB Enhancement by PADK for Synaptic and Functional Improvements in Aged Mice

We used 22-month-old C57BL/6 mice as another model of age-related cognitive impairment [42] in case extended aging elicits cellular risk factors related to the continuum from MCI to AD dementia. To test the noted therapeutic strategy as a means to offset age-related lysosomal perturbation, PADK was administered orally in order to avoid the stress of i.p. injections in 22-month mice. Firstly, the compound was determined to be orally bioavailable in adult rats after 11 days of dosing every 12 h, with PADK detected at 21–49 ng/g brain tissue across dosages tested (Center for Drug Discovery mass spectrometry). The treated rats exhibited a dose-dependent increase in the active CatB isoform (Figure 6F; Kruskal–Wallis test: *p* = 0.0006) as well as for enhanced CatB proteolytic activity (Figure 6G; Kruskal–Wallis test: *p* = 0.0041) Next, the 22-month C57BL/6 mice received ZFA or PADK via oral administration twice daily for 11 days to assess behavior and the GluR1 synaptic marker. As found in the Fischer rats, the age-related compromise of GluR1 in the C57BL/6 mice was significantly restored in correspondence with CatB modulation (Figure 6H,I) as was performance levels for exploratory habituation (Figure 6J). Young mice performed with the normal behavior of reduced exploration on the second day of exposure to a novel open field environment (data in red; *p* = 0.0156 by nonparametric analysis), whereas the 22-month mice did not. Changes in the behavior were evident in the aged mice across exploration days and treatment type (blue data; Kruskal–Wallis test: *p* = 0.0115), with only the PADK-treated mice displaying habituated exploration on day two, similar to the young. No differences in gross mobility were found across the four assessment datasets (Figure 6K). Synaptophysin immunostaining in the mouse brains (Figure 6L) further supports the evident link between improved behavioral performance and restoration of synaptic integrity in key neuronal networks.

## 3. Discussion

The current study indicates that lysosomes are intimately involved in multiple types of age-related disorders. Abnormal lysosomal immunolabeling was associated with amyloid plaques formed through Aβ deposition, and proteolytic detoxification of the Aβ42 peptide was localized to neuronal lysosomes, thus further adding to the substantial support for their role in Aβ pathology. In addition to AD pathogenesis, our finding also link aspects of Parkinson’s disease and MCI to lysosomal disturbances. Furthermore, enhancing a component of the lysosomal pathway reduced several indications of protein accumulation pathology, again pointing to the vital role lysosomes play in maintaining cellular homeostasis. The positive modulation of the lysosomal CatB enzyme led to a wide range of influences in the different animal models tested, including reduced amyloidogenic peptide species and protection against early α-synucleinopathy, as well as synaptic and functional improvements in models of age-related cognitive impairment. Being a major part of the autophagy–lysosome system, lysosomal alterations likely contribute to the age-related dysfunction in autophagic–lysosomal processing, consisting of impairment in organelle maturation and fusion with autophagosomes.

The results from APP/PS1 brain tissue with evident synaptopathology described amyloid deposits surrounded by swollen lysosomal structures containing the LAMP1 marker. The altered positioning of enlarged lysosomes was more pronounced around larger plaques, with the colocalization well evident in brain regions known to exhibit vulnerability in AD. The overall findings further substantiate those of many important studies using AD tissue and experimental models with regard to lysosomes and their link to the neurodegenerative disorder [4,33,43,44,45,46]. In AD brains, reported changes include altered levels of CatB-containing neuronal lysosomes and impairment in autophagic steps involving lysosomes [20,47]. Abnormal positioning of lysosomes and their markers in close proximity to amyloid plaques was described in AD and mouse models [33,44,47], perhaps related to lysosomal accumulation of amyloidogenic material that is also linked to synaptic pathology [35,48,49]. It should be noted that APP-βCTF accumulation was implicated in lysosomal dysfunction independent of Aβ production [35,50], and that endosomal–lysosomal dysregulation was found in MCI independent of Aβ pathology [51]. Interestingly, the APP-βCTF-producing β-secretase was also found enriched at amyloid plaques, and found to colocalize with the LAMP1 lysosomal marker [33]. The significant Gowrishankar et al. study identified dystrophic axons accounting for most of the plaque-localized lysosomes deemed as protease-deficient, and such axonopathy initiated at early plaque formation, involving local impairment of retrograde transport and lysosome precursors affected by disrupted maturation. Additional studies point to transport failure as a key component of the gradual pathogenic cascade underlying protein accumulation stress that leads to synaptic deterioration [52,53,54], the best correlate of cognitive decline [55,56,57].

By targeting the lysosomal protein clearing function in a way to offset impairments to lysosomes in APP/PS1 mice, the positive lysosomal modulator PADK resulted in enhanced levels of active CatB and improved proteolytic truncation of Aβ42. Detoxification of the Aβ42 peptide to smaller, less amyloidogenic species coincided with grouped assemblies of lysosomes within neurons. The altered polar positioning of neuronal lysosomes involved apparent clusters conducting Aβ42 detoxification, perhaps related to a report indicating that lysosomal positioning influences anabolic and catabolic responses including autophagosome–lysosome fusion [58]. The PADK modulator treatment also promoted colocalization of Aβ42 with the Aβ42-degrading hydrolase CatB as previously found [1]; note that PADK was reported to disrupt Aβ42 oligomeric states [59] as a potential additive influence that facilitates monomeric peptide transfer to lysosomes. Such influences on aspects of the autophagy–lysosomal protein clearance pathway are important in light of the fact that deficiencies in the pathway constitute a major factor in AD and other proteinopathies [1,4,60,61].

Enhancement of the active isoform of CatB strongly correlated with improved CatB activity to explain the reduction in intracellular Aβ42 and increased levels of Aβ38, with this Aβ42 detoxification also corresponding with clearance of extracellular deposits. Regarding another amyloidogenic peptide, the APP-βCTF species found in different disorders [35,50,62], it was also significantly reduced in PADK-treated APP/PS1 mice in close correspondence with indicators of synaptic, neuropilar, and behavioral restoration. Positive modulation of CatB was previously shown to elicit synaptic protection in multiple types of AD mouse models [13,14,15,63,64] and in brain explant models of AD-type protein accumulation pathology [12,16,54,65]. Across the group of small-molecule CatB-enhancing compounds identified, a common feature was their ability to protect synapses during protein accumulation stress. As the current transgenic mouse studies found CatB enhancement linked to reduced Aβ pathology and improved synaptic and cognitive measures, it is also relevant to point out a study showing that aspirin induces lysosomal biogenesis in vitro with a corresponding increase in CatB activity, as well as in 5× familial AD mice with corresponding reductions in APP-βCTF and Aβ peptides [66]. While there are no indications that PADK induces lysosome biogenesis [14], it cannot be ruled out whether the lysosomal modulator has a positive influence on other mechanistic avenues including (i) endolysosomal protein trafficking shown to reduce Aβ deposition and restore synaptic integrity in APP/PS1 mice [67], (ii) proficiency of lysosomal membrane recycling [68], or (iii) the efficiency of lysosomal fusion with multivesicular bodies, related protein sequestration, or delivery/release of extracellular vesicles [69,70,71].

The PADK treatment also influenced human α-synuclein levels in transgenic mice expressing A53T α-synuclein, a human variant linked to familial Parkinson’s disease. Note that α-synuclein is a major component of intracellular proteinaceous inclusions found in several neurodegenerative disorders. As with APP/PS1 mice, CatB-enhancing PADK treatment in animals with early α-synucleinopathy led to an evident link between pathogenic protein reduction and improved synaptic marker levels. These findings corroborate with studies pointing to lysosomal dysfunction as playing a part in Parkinson’s disease [72,73,74] and with those indicating involvement of α-synuclein aggregates in altering dendritic spines and disrupting synaptic activity before cell death in excitatory hippocampal neurons [75]. Positive lysosomal modulation was previously suggested as a therapeutic approach for the protein accumulation stress associated with Parkinson’s disease [76,77,78], and restoring lysosomal levels was reported to attenuate cell death induced by a parkinsonian neurotoxin in vitro and to reduce dopaminergic neurodegeneration in vivo [74]. Whether PADK-induced α-synuclein clearance is mediated by CatB or another mechanism is unclear; thus, it cannot be ruled out whether the lysosomal modulation involves the altering of lysosomal exocytosis. Impairment of the lysosomal exocytosis pathway by gene mutations linked to Parkinson’s disease was recently found to contribute to α-synuclein accumulation in dopaminergic neurons, and upregulating the pathway rescued such accumulation in patient neurons [79].

Lastly, deficits in synaptic markers and learning were offset in non-amyloid models of MCI following lysosomal enhancement treatment. Assessments regarding age-related cognitive impairment are important since MCI likely represents a precursor to AD [39,40,41,80], with parallel implications of oxidatively modified proteins identified in AD, MCI, and Aβ models [81], thus inferring that gradual protein accumulation stress underlies the risk of age-related progression toward dementia. With the evidence that compensatory lysosomal responses involve CatB to offset protein accumulation pathology [13,14,15,63,64], it should be pointed out that CatB in blood plasma appeared to gradually increase across the stages of MCI, mild AD, and severe AD [24], suggesting a progressive CatB response as protein accumulation pathology worsens. Note that, in healthy individuals, the extent of increased plasma CatB through exercise correlated with improved learning and memory [25], and, in a mouse model of Parkinson’s disease, endurance exercise increased autophagy and cathepsin proteins, as well as reduced α-synuclein levels, improved dopaminergic markers, and restored motor function [82]. In the models of MCI utilized in the current study, the CatB enhancement by PADK led to evidence of recovered levels of the GluR1 AMPA receptor subunit, occurring in correspondence with improved learning and memory. Interestingly, AMPA receptor activity promotes the stabilization and dendritic spine-localized clustering of drebrin [83], a synaptic protein significantly reduced in the hippocampus of both MCI and AD individuals [41,83].

Together, the findings in this study indicate that lysosomal dysfunction contributes to the synaptopathology associated with AD, potential precursors of AD, and other types of proteinopathy. The fact that different types of protein accumulation pathology are offset by enhancing a protein clearance mechanism of lysosomes clearly points to the broad importance for lysosomal organelles with regard to proteostasis and synaptic maintenance. With synapse loss being the best correlate concerning the progression of cognitive decline, the early CatB modulation is deemed a potential avenue to reduce risk factors and slow the gradual pathogenesis that leads to dementia. As emphasized in a recent and extensive review [84], therapeutically targeting lysosomal proteins in a specific manner could emerge with applications encompassing neurodegenerative and metabolic diseases, as well as senescence and aging.

## 4. Materials and Methods

### 4.1. Animal Models

All use of animals adhered to recommendations from the Guide for the Care and Use of Laboratory Animals of the National Institutes of Health of the United States, and this study was carried out in strict accordance with the Animal Welfare Act and other federal statutes and regulations related to animals and their ethical use. The research was conducted in accordance with approved protocols of the Animal Care and Use Committees of the University of North Carolina–Pembroke (Protocols 2013-01-2 and 2018-02) and of the University of Connecticut (Protocol BAB-008). All animals were housed in vivarium facilities until the desired age and, when necessary, genotyping was confirmed by PCR on tail DNA. Transgenic mice and their non-transgenic littermates were from Jackson Laboratories (Bar Harbor, Maine, USA), including APPswe/PS1ΔE9 mice (APP/PS1), strain B6, used at 22–23 months of age, and A53T transgenic mice expressing A53T human α-synuclein, used at 6–7 months of age.

Aged rodents used for age-related MCI models were obtained at least 1–2 months before experimental treatments. Animals were kept in a humidity-controlled and temperature-controlled room until the desired age. Different groups of aged mice (C57BL/6) were obtained from the National Institute of Aging rodent colonies and were used at 8–22 months of age and compared to young mice. Male Sprague-Dawley rats and female Fischer rats were obtained from Charles River Laboratories (Wilmington, Massachusetts, USA) and used at 11 months old.

### 4.2. Treatments

For the daily injections of APP/PS1, A53T, and corresponding wild-type mice, Z-Phe-Ala-diazomethylketone (PADK), routinely obtained from Bachem Americas, Inc. (Torrance, California, USA), was initially prepared at 24 mg/mL in dry dimethyl sulfoxide (DMSO), and slowly diluted with phosphate-buffered saline (PBS) to 12 mg/mL. Before the injections, the animals were handled daily over a week period, and, during a period of 11 days, each animal received i.p. injections of 18 mg/kg PADK or the corresponding volume of vehicle (50% PBS and 50% DMSO). Fischer rats received daily i.p. injection of 27 mg/kg PADK or the corresponding volume of vehicle over a period of nine days. Nonpeptidyl CatB-enhancing compounds were administered at 20–25 mg/kg/day.

For oral dosing, compounds were added to food discs made from peanut butter following a procedure adapted from Gonzales et al. [85]. The PADK compound or the structurally similar but inactive compound ZFA (Bachem Americas, Inc.) was added to the mixture for the desired dosage, and the food discs were stored at −80 °C with appropriate amounts periodically thawed for treatments administered twice daily. Animals were observed to ensure consumption of each treatment amount, which was determined by the daily animal weight.

For tests of CatB modulation in human neurons, human iPSC-derived neurons (Cellular Dynamics International; Madison, Wisconsin, USA) were maintained in culture for 14 days followed by treatment with ZFA or PADK. After three daily treatments, the neurons were assessed for CatB.

### 4.3. Behavioral Analyses

One day before the end of the compound administration period, APP/PS1 and wild-type mice were assessed by a series of behavioral paradigms. Following the prior handling and familiarization with each apparatus, the mice were tested for spontaneous alternation behavior using a T-maze as previously described [14,86]. For the T-maze test, mice were placed at the intersection of a T-maze, and entries across each arm’s threshold were observed with a closed-circuit monitor for a 10-min period. A minimum of 15 entries was used to determine percent alternations when compared to total alternations possible. An alternation was a succession of entries into three different arms of the maze. Additional analyses included the rotarod test to measure the time maintaining coordinated movement on a 15-rpm rotating rod. A modified paradigm of conditioned place preference (CPP) was used to assess time to enter the specific compartment previously trained over 10 trials as the reward location.

The female Fischer rats were assessed for passive avoidance over a three-day schedule using an apparatus with dark and light compartments connected by a gate. Briefly, on day one (pre-shock), the rats were allowed to explore both compartments and number of entries and time spent in each were recorded. On day two (shock), rats were administered a minor foot shock immediately upon entry into the dark compartment and subsequent gate closure. On day three (post-shock), each rat was placed in the light compartment with free access to the dark compartment, and the number of entries into the latter and time spent there were recorded.

The aged mice of 22 months were tested to determine exploratory distance and rate of mobility during novel exposure to an open-field environment, with grid crossings assessed over a 5-min period using a closed-circuit camera. Subsequent re-exposure to the open field was conducted 24 h later in order to calculate exploratory habituation (percentage change from first day’s exploration).

### 4.4. Sample Preparation for Analyses

Following behavioral tests, brain tissue was rapidly removed and prepared for analyses. To perform the hematoxylin–eosin staining and immunofluorescence protocols, a subset of animals from each condition were anesthetized and perfused with 4% paraformaldehyde prior to tissue dissection and sectioning. All other brains were removed and dissected in ice-cold buffer containing 0.32 M sucrose, 5 mM 4-(2-hydroxyethyl)-1-piperazineethanesulfonic acid (HEPES; pH 7.4), 1 mM ethylenediaminetetraacetic acid (EDTA), 1 mM ethylene glycol-*bis*(2-aminoethyl ether)-*N*,*N*,*N*’,*N*’- tetraacetic acid (EGTA), and the protease inhibitors aprotinin, leupeptin, bestatin, E-64, pepstatin A (each at 2 µg/mL), and 4-(2-aminoethyl) benzenesulfonyl fluoride (0.3 mM) (reagents from Sigma-Aldrich, St. Louis, Missouri, USA), followed by snap-freezing of sample aliquots in liquid nitrogen, later thawed for homogenization steps. The protease inhibitors were absent from solutions needed to dissect brains from the subsets of mice and rats used for multiple studies through immunoblot analyses, protease activity measurements, and messenger RNA (mRNA) analyses. The latter were conducted by Jinfiniti Biosciences (Augusta, Georgia, USA) using iNtRON Biotechnology miRacle RNA extraction kits to prepare total RNA from hemibrains, which was reverse-transcribed to complementary DNA (cDNA) for qRT-PCR analyses in duplicate with an Applied Biosystems HT7900 and Taqman assays specific to the rodent CatB and β-actin genes.

### 4.5. Immunoblot Analysis

Protein contents of homogenized samples were determined with a colorimetric protein assay and a bovine serum albumin (BSA) standard curve, and equal protein amounts of compared samples were denatured for 5 min at 100 °C, separated by SDS-PAGE, and transferred to nitrocellulose membranes for immunolabeling procedures as previously described [49,87,88]. Antibodies utilized were developed against cathepsin B (1:200; Millipore; Danvers, Massachusetts, USA), β-actin (1:1000; Sigma-Aldrich), amino acids 1–16 of human Aβ (6E10, 1:500; Covance; Princeton, New Jersey, USA), human sAPPα (2B3, 1:100, IBL International; Morrisville, North Carolina, USA), AMPA receptor subunit GluR1 [89] (1:300; or 1:1000 when supplied from Millipore), NCAM-180 (1:300; Abcam), human α-synuclein (1:200; Abcam), and SRPX2 (1:300; Proteintech Group, Inc., Rosemont, Illinois, USA). The latter is the sushi repeat-containing protein X-linked 2 that interacts with CatB [90]. Secondary antibodies were from Bio-Rad and immunoreactive bands were assessed for integrated optical density with BIOQUANT software 8.4 (BIOQUANT Image Analysis Corporation; Nashville, Tennessee, USA). The gel load control was typically β-actin being assessed in blot sections or after stripping immunoblots subsequent to labeling synaptic markers. Alternative load-control antigens included tubulin and other cytoskeletal proteins of varying molecular weights.

### 4.6. ELISA

Detection of Aβx-42 and Aβx-38 peptide species was assessed by two sandwich ELISA protocols. Equal protein aliquots of solubilized homogenate samples were applied to microtiter plates containing antibodies that specifically recognize Aβx-42 (12F4, Covance; Princeton, New Jersey, USA) or Aβx-38 (BA1–13, Covance). Chemiluminescence detection of captured peptides was measured with a SpectraMax L Luminescence Reader (Molecular Devices; Sunnyvale, California, USA) and converted to femtomoles per milligram sample protein using standard curves generated with pure Aβ42 and Aβ38 peptides (Bachem Americas, Inc.; Torrance, California, USA).

### 4.7. Immunohistochemistry

Fixed tissue was cryoprotected and serially sectioned at a thickness of 10–20 μm. The tissue sections were immunolabeled using 6E10 antibody (Covance), anti-calbindin D28k (Sigma-Aldrich), anti-GluR1 (from Millipore or developed as described [88]), anti-LAMP1 (BD Pharmingen; San Jose, California, USA), anti-cathepsin B (Millipore), anti-synaptophysin (Millipore), and antibodies that selectively label Aβ38 and Aβ42 (Covance). Immunofluorescence analyses used appropriate Invitrogen secondary antibodies (Thermo Fisher Scientific; Waltham, Massachusetts, USA), and images were captured with a Zeiss fluorescence microscope system (Carl Zeiss, Inc.; Thornwood, New York, USA) and with a Nikon C2 point-scanning confocal microscope with NIS-Elements AR software (Nikon Instruments; Melville, New York, USA). Other images were produced via avidin–biotin–peroxidase protocols (Vector Laboratories; Burlingame, California, USA) that used 3,3′-diaminobenzidine as the chromogen. In each case, treatment groups were immunostained together and analyzed under the same instrument settings. Equally spaced coronal sections along the rostral–caudal axis of the hippocampus were used to determine the average immunoreactivity intensity and area of staining across four different view-fields.

### 4.8. Cathepsin B Activity and Proteasome Activity

CatB enzymatic activity was assessed in brain sample aliquots using the InnoZyme Cathepsin B Activity Assay Kit (Millipore) as previously described [14,27]. Equal amounts of brain homogenates (10 μg of protein) were assessed in duplicate per sample for proteolytic activity using the Z-Arg-Arg-7-amido-4-methylcoumarin (AMC) substrate and a SpectraMax M3 microplate reader. To confirm specific measuring of CatB activity, the CatB inhibitor CA074 (10 μM) was added to a subset of samples to determine that the measured substrate cleavage was mediated by CatB. For proteasome activity, the suc-Leu-Leu-Val-Tyr-AMC substrate (Millipore) was utilized in order to determine cleavage in freshly prepared tissue samples of equal protein aliquots as previously described [12].

### 4.9. Statistical Analysis

The quantitative results were evaluated by unpaired, Mann–Whitney nonparametric tests, Kruskal–Wallis nonparametric tests across treatment groups, Dunn’s multiple comparison post hoc tests, Wilcoxon tests, linear regression analyses, and χ^2^ analyses of categorical distributions using GraphPad Prism 7 (GraphPad Software, San Diego, California, USA).

## 5. Patents

This research supported United States (US) Patent 8,163,953 (title: Compounds for lysosomal modulation and methods of use—for treating Alzheimer’s disease, Parkinson’s disease, Huntington’s disease, and amyotrophic lateral sclerosis).

## Figures and Tables

**Table 1:** Small-molecule cathepsin B (CatB)-enhancing compounds promote synaptic recovery. PADK—Z-Phe-Ala-diazomethylketone; GluR1—glutamate receptor 1.

Compound	Molecular Weight (g/mol)	CatB Change	GluR1 Recovery
PADK	394.4	4.9-fold increase	100%
SD1002	446.5	3.1-fold increase	100%
BW1005	496.5	2.8-fold increase	100%
UP1A101	328.4	2.1-fold increase	53%
E64d	342.4	1.7-fold increase	39%

Compounds were assessed for altering CatB levels in hippocampus compared to vehicle treatment, as well as for GluR1 recovery in models of protein accumulation pathology.

**Table 2:** Assessments related to selectivity, safety, and gender for CatB-enhancing compound PADK. LAMP1—lysosome-associated membrane protein 1; mRNA—messenger RNA; SRPX2—sushi repeat-containing protein X-linked 2.

Measurements	Results Determined after PADK Treatment
LAMP1-positive organelles/neuron	97 ± 9% of vehicle-treated mice (N.S.)
CatB-positive organelles/neuron	98 ± 5% of vehicle-treated mice (N.S.)
CatB mRNA in transgenic mice	−0.497 ΔΔC*t* compared to vehicle-treated mice (N.S.)
CatB mRNA in Fischer rats	−0.599 ΔΔC*t* compared to vehicle-treated rats (N.S.)
Proteasome activity	117 ± 12% of vehicle-treated controls (N.S.)
SRPX2 CatB-interacting protein	95 ± 6% of vehicle-treated controls (N.S.)
Body weight of 9-month female mice	103.7 ± 1.9% of start weight (N.S.)
Body weight of 9-month male mice	104.9 ± 1.4% of start weight (N.S.)
Body weight of 22-month male mice	101.8 ± 0.6% of start weight (N.S.)

N.S., not significant as per nonparametric tests.

## Supplementary Materials

Supplementary materials can be found at https://www.mdpi.com/1422-0067/20/18/4432/s1.

## Abbreviations

act Cat B	30-kDa active cathepsin B
*Actb*	β-actin gene
AD	Alzheimer’s disease
APP	amyloid precursor protein
Cat B	cathepsin B
CTF	carboxyl terminal fragment
CPP	conditioned place preference
DG	dentate gyrus
H&E	hematoxylin and eosin
iPSC	induced pluripotent stem cell
LAMP1	lysosomal-associated membrane protein 1
MCI	mild cognitive impairment
mf	mossy fibers
ml	molecular layer
NCAM	neural cell adhesion molecule
PADK	Z-Phe-Ala-diazomethylketone
PCR	polymerase chain reaction
PS1	presenilin 1
qRT-PCR	real-time quantitative reverse transcription PCR
SAB	spontaneous alternation behavior
sg	stratum granulosum
so	stratum oriens
sp	stratum pyramidale
veh	vehicle
WT	wild type
yg	young
ZFA	Z-Phe-Ala-OH
