# Supplementary material for: The Role of Lysosomes in a Broad Disease-Modifying Approach Evaluated across Transgenic Mouse Models of Alzheimer’s Disease and Parkinson’s Disease and Models of Mild Cognitive Impairment
Source: Int J Mol Sci. 2019 Sep 9;20(18):4432. doi: 10.3390/ijms20184432 (PMC6770842; doi:10.3390/ijms20184432)

Supplementary Information

The Role of Lysosomes in a Broad Disease-Modifying Approach Evaluated across Transgenic Mouse Models of Alzheimer's Disease and Parkinson's Disease and Models of Mild Cognitive Impairment

Jeannie Hwang [1,2], Candice M. Estick [2,3], Uzoma S. Ikonne [1], David Butler [2,4 *], Morgan C. Pait [1,5], Lyndsie H. Elliott [1,6], Sarah Ruiz [1,6], Kaitlan Smith [1,7], Katherine M. Rentschler [1], Cary Mundell [1,6], Michael F. Almeida [1], Nicole Stumbling Bear [1,6], James P. Locklear [1,6], Yara Abumohsen [1,6], Cecily M. Ivey [1,6],, Karen L.G. Farizatto [1,6], and Ben A. Bahr[1,2,5-7 *]

[1]William C. Friday Laboratory, Biotechnology Research and Training Center, University of North Carolina– Pembroke, Pembroke, North Carolina 28372 U.S.A.
[2]Department of Pharmaceutical Sciences and the Neurosciences Program, University of Connecticut, Storrs, Connecticut 06269 U.S.A.
[3]Department of Physiology and Neurobiology, University of Connecticut, Storrs, Connecticut 06269 U.S.A.
[4]Center for Drug Discovery, Northeastern University, Boston, Massachusetts 02115 U.S.A.
[5]Department of Chemistry and Physics, University of North Carolina–Pembroke, Pembroke, North Carolina 28372 U.S.A.
[6]Department of Biology, University of North Carolina–Pembroke, Pembroke, North Carolina 28372 U.S.A.
[7]Molecular Biotechnology Program University of North Carolina–Pembroke, Pembroke, North Carolina 28372 U.S.A.

***Corresponding author:**
B.A. Bahr, Biotech Center and Training Center,
1 University Drive, University of North Carolina – Pembroke, Pembroke NC 28372
Email: Bahr@uncp.edu

Hwang et al., supplementary information for Figure 2A

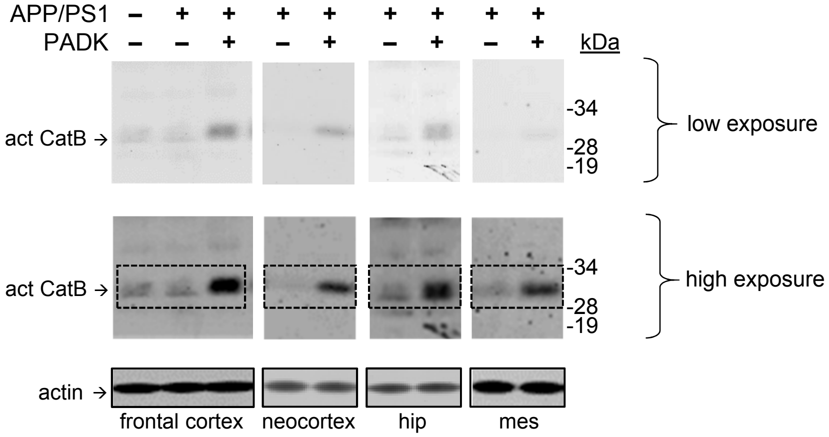

Hwang et al., supplementary information for Figure 3A

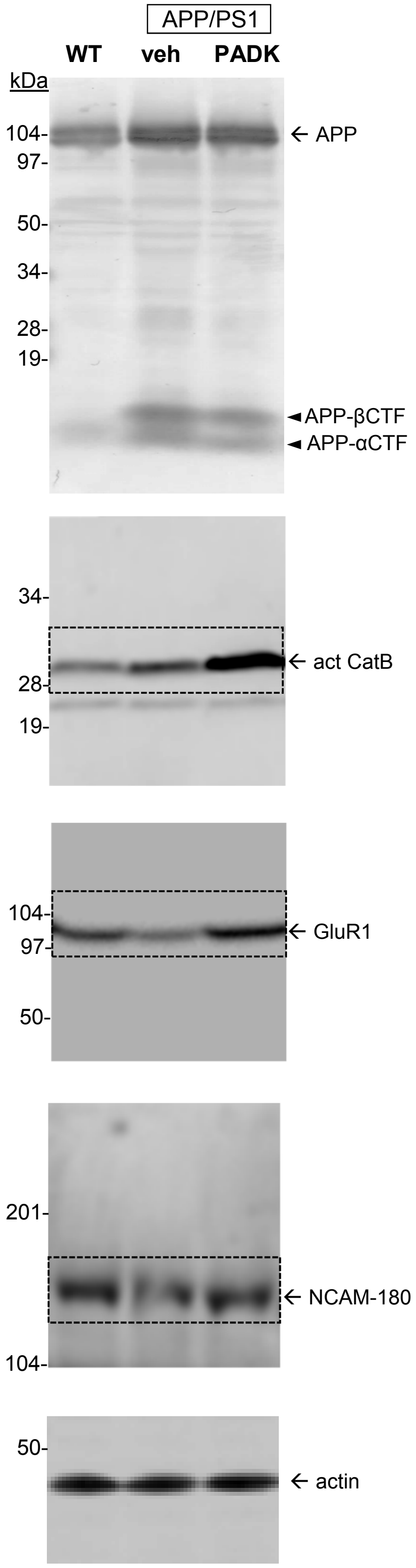

Hwang et al., supplementary information for Figure 3B

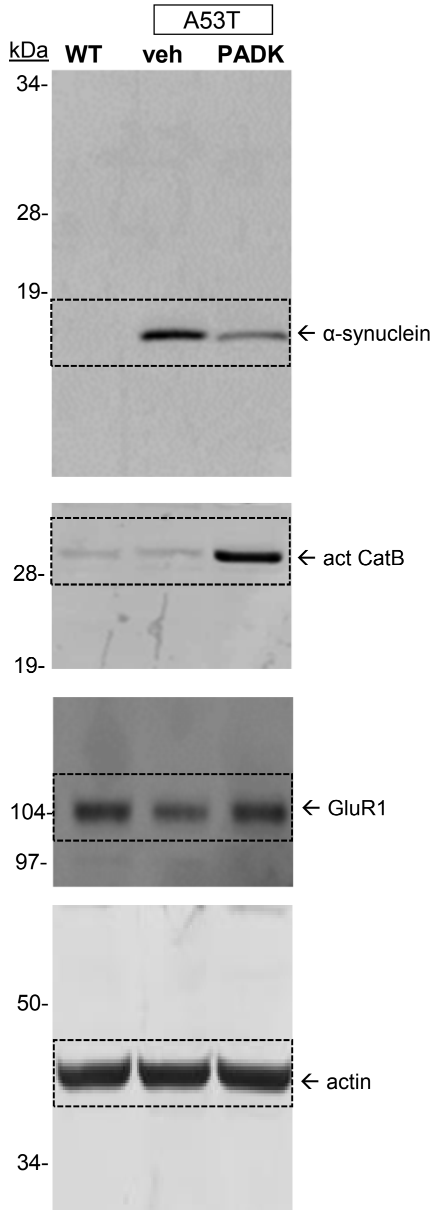

Hwang et al., supplementary information for Figure 6A

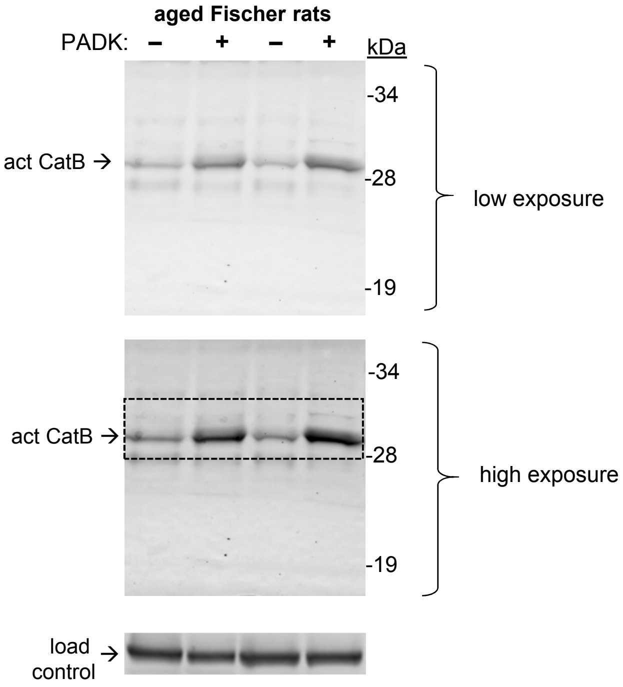

Hwang et al., supplementary information for Figure 6C

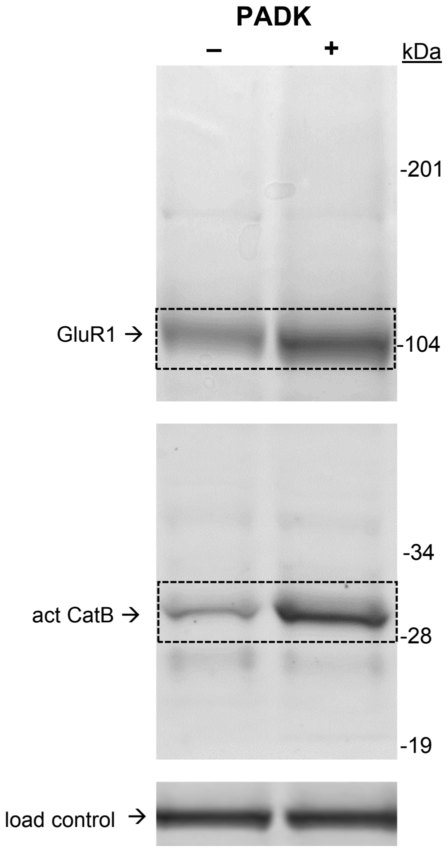

Hwang et al., supplementary information for Figure 6E

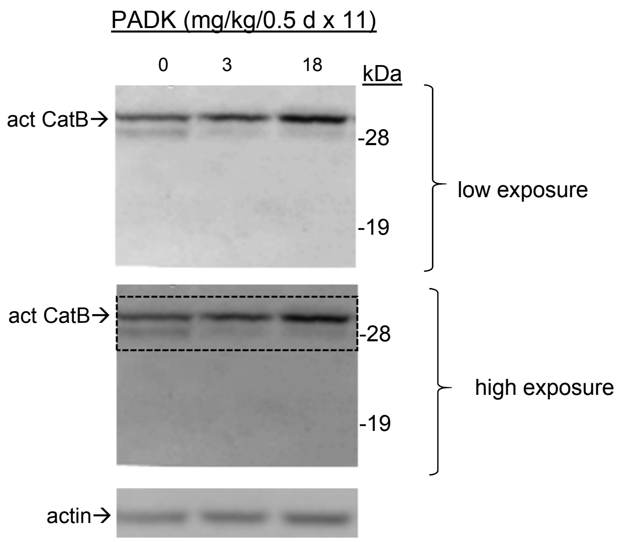

Hwang et al., supplementary information for Figure 6H

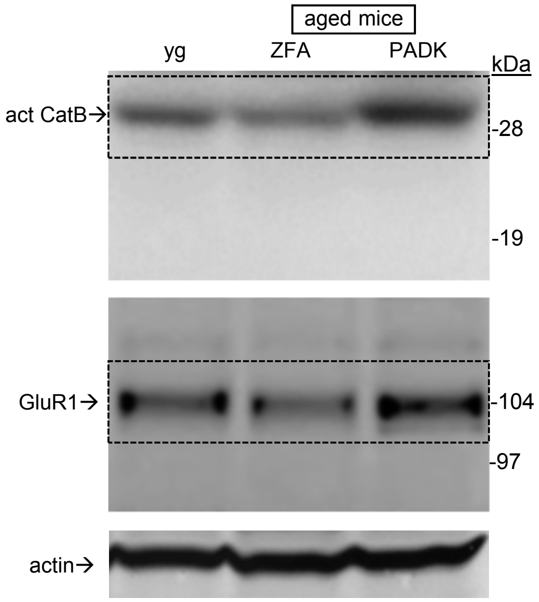

Supplement: Supplementary file 1 [file ijms-20-04432-s001.pdf]
